# Efficacy and safety of abatacept or infliximab vs placebo in ATTEST: a phase III, multi-centre, randomised, double-blind, placebo-controlled study in patients with rheumatoid arthritis and an inadequate response to methotrexate

**DOI:** 10.1136/ard.2007.080002

**Published:** 2007-11-29

**Authors:** M Schiff, M Keiserman, C Codding, S Songcharoen, A Berman, S Nayiager, C Saldate, T Li, R Aranda, J-C Becker, C Lin, P L N Cornet, M Dougados

**Affiliations:** 1 Denver Arthritis Clinic, Denver, Colorado, USA; 2 Pontificial Catholic University, School of Medicine, Porto Alegre, Brazil; 3 Health Research of Oklahoma, Oklahoma City, Oklahoma, USA; 4 Arthritis & Osteoporosis Center, Flowood, Missouri, USA; 5 Centro Medico Privado De Reumatologia, Tucuman, Argentina; 6 St Augustine’s Hospital, Durban, South Africa; 7 Centro de Investigacion del Noroeste, Tijuana, Mexico; 8 Bristol-Myers Squibb, Princeton, New Jersey, USA; 9 Bristol-Myers Squibb, Pennington, New Jersey, USA; 10 Paris-Descartes University, Medicine Faculty and UPRES-EA 4058; AP-HP, Cochin Hospital; Paris, France

## Abstract

**Objectives:**

This double-blind trial evaluated the efficacy and safety of abatacept or infliximab vs placebo. The primary objective of this study was to evaluate the mean change from baseline in Disease Activity Score (based on erythrocyte sedimentation rates; DAS28 (ESR)) for the abatacept vs placebo groups at day 197.

**Methods:**

Patients with rheumatoid arthritis (RA) and an inadequate response to methotrexate (MTX) were randomised 3:3:2 to abatacept (∼10 mg/kg every 4 weeks, n = 156), infliximab (3 mg/kg every 8 weeks, n = 165), or placebo (every 4 weeks, n = 110) and background MTX. Safety and efficacy were assessed throughout the study.

**Results:**

Similar patient demographics and clinical characteristics were present at baseline between groups, with mean scores of ∼1.7 for HAQ-DI and 6.8 for DAS28 (ESR). At 6 months, mean changes in DAS28 (ESR) were significantly greater for abatacept vs placebo (–2.53 vs –1.48, p<0.001) and infliximab vs placebo (–2.25 vs –1.48, p<0.001). For abatacept vs infliximab treatment at day 365, reductions in the DAS28 (ESR) were –2.88 vs –2.25. At day 365, the following response rates were observed for abatacept and infliximab, respectively: American College of Rheumatology (ACR) 20, 72.4 and 55.8%; ACR 50, 45.5 and 36.4%; ACR 70, 26.3 and 20.6%; low disease activity score (LDAS), 35.3 and 22.4%; DAS28-defined remission, 18.7 and 12.2%; good European League Against Rheumatism (EULAR) responses, 32.0 and 18.5%; and Health Assessment Questionnaire Disability Index (HAQ-DI), 57.7 and 52.7%. Mean changes in physical component summary (PCS) were 9.5 and 7.6, and mental component summary (MCS) were 6.0 and 4.0, for abatacept and infliximab, respectively. Over 1 year, adverse events (AEs) (89.1 vs 93.3%), serious AEs (SAEs) (9.6 vs 18.2%), serious infections (1.9 vs 8.5%) and discontinuations due to AEs (3.2 vs 7.3%) and SAEs (2.6 vs 3.6%) were lower with abatacept than infliximab.

**Conclusions:**

In this study, abatacept and infliximab (3 mg/kg every 8 weeks) demonstrated similar efficacy. Overall, abatacept had a relatively more acceptable safety and tolerability profile, with fewer SAEs, serious infections, acute infusional events and discontinuations due to AEs than the infliximab group.

**Trial registration number:**

NCT00095147.

Abatacept is a selective T-cell co-stimulation modulator, that modulates the CD80/CD86:CD28 co-stimulatory signal required for full T-cell activation.^1^ The mechanism of action of abatacept is fundamentally different to that of other biological disease-modifying antirheumatic drugs (DMARDs) for the treatment of rheumatoid arthritis (RA). The efficacy of abatacept has previously been demonstrated in patients with RA and an inadequate response to methotrexate (MTX)^2^ and anti-tumour necrosis factor (TNF) agents,^3^ respectively.

The ATTEST (for “Abatacept or infliximab vs placebo, a Trial for Tolerability, Efficacy and Safety in Treating rheumatoid arthritis”) trial was designed to obtain data on the magnitude of the treatment effect in RA of abatacept or infliximab (an established inhibitor of TNF for RA) vs placebo, and to obtain relative efficacy and safety data on these two biological treatments in a single study. The study utilised a double-blind, randomised, placebo-controlled design for the first 6 months to validate efficacy responses, and the study duration allowed for the opportunity to directly compare the safety profile of the active biologic treatment groups over 1 year.

## METHODS

### Patients

Eligible patients met the American College of Rheumatology (ACR) criteria for RA, were at least 18 years of age, had RA for at least 1 year,^4^ and had an inadequate response to MTX, as demonstrated by ongoing active disease (at randomisation ⩾10 swollen joints, ⩾12 tender joints, and C-reactive protein (CRP) levels ⩾1 mg/dl using a high sensitivity assay (upper limit of the normal range, 0.5)). All patients had received MTX ⩾15 mg/week for ⩾3 months prior to randomisation (stable for at least 28 days) and washed out all DMARDs (⩾28 days prior) except for MTX. No prior experience of abatacept or anti-TNF therapy was permitted. All patients were screened for tuberculosis (TB) by purified protein derivative (PPD) testing and chest *x* ray. The protocol used for TB screening was the same as that employed in the “Anti-TNF Trial in rheumatoid arthritis with Concomitant Therapy” (ATTRACT) trial.^5^

Concomitant medications were permitted between days 1–197: oral corticosteroids (⩽10 mg of prednisone or equivalent daily (stable for ⩾25 out of 28 days prior to randomisation)), and/or stable non-steroidal anti-inflammatory drugs (NSAIDs) including acetyl salicylic acid, and analgesics not containing aspirin or NSAIDs. No MTX dose adjustments were permitted except in the occurrence of adverse events (AEs). Between days 198–365, dose modification was permitted for MTX (⩽25 mg weekly) and oral corticosteroids (⩽10 mg prednisone or equivalent daily); hydroxychloroquine, sulfasalazine, gold, or azathioprine were also permitted. Premedication prior to infusions of study drug was left at the discretion of the investigator (not required by protocol).

### Study design

ATTEST was a randomised, double-blind, double-dummy, placebo- and active (infliximab)-controlled, 12-month global trial. Adult patients with active RA and an inadequate response to MTX were randomised by centre in a 3:3:2 ratio to 6 months of abatacept (approximating 10 mg/kg), infliximab (3 mg/kg), or placebo treatment by intravenous (IV) infusion, on a background of MTX. Assessors, physicians and patients were blinded to the treatment group assignment for 1 year.

The study was approved by the institutional review boards and independent ethics committees at participating sites, and was carried out in accordance with the ethical principles of the Declaration of Helsinki. All patients provided written informed consent before randomisation.

Treatment with placebo was limited to days 1–197 to provide internal validity to the trial design and the clinical response rates of the two active treatment groups. On day 198, placebo-treated patients were reallocated to abatacept (with blinding maintained). Patients initially randomised to abatacept or infliximab continued their treatment. Clinical efficacy and safety assessments are presented for the abatacept, placebo and infliximab groups up to day 197, and for the abatacept and infliximab groups up to day 365 (excluding patients in the placebo group who were reallocated to abatacept at day 198).

### Study drugs

Abatacept was dosed according to weight: patients weighing less than 60 kg, 60–100 kg, or more than 100 kg received 500 mg, 750 mg, or 1000 mg of abatacept, respectively. Infliximab was dosed at 3 mg/kg for all patients. Abatacept was administered by IV infusion on days 1, 15 and 29, and every 28 days thereafter, up to and including day 337 (with normal saline received on day 43). Infliximab was administered on days 1, 15, 43 and 85, and every 56 days thereafter (normal saline was received at the remaining visit days). Two IV bags were infused simultaneously to ensure blinding to treatment group assignment, one over 30 min (abatacept or placebo) and one over 2 h (infliximab or placebo).

### Objectives

The primary endpoint was to evaluate a reduction in disease activity, measured by Disease Activity Score 28 (based on erythrocyte sedimentation rate levels; DAS28 (ESR)) with abatacept vs placebo at 6 months. Secondary endpoints included mean reduction in DAS28 (ESR) with infliximab vs placebo at 6 months. Additional secondary endpoints at 6 months and 1 year included: mean reduction in DAS28 (ESR) with abatacept vs infliximab; DAS28 (ESR) European League Against Rheumatism (EULAR) responses;^6^ low disease activity score (LDAS; DAS28 (ESR) ⩽3.2); DAS28 (ESR)-defined remission (DAS28 (ESR) <2.6); ACR 20, 50 and 70 responses;^7^ Health Assessment Questionnaire Disability Index (HAQ-DI) response rates (⩾0.3 improvement from baseline); and mean changes in the physical and mental component summary (PCS and MCS, respectively) scores, and eight subscales of the Short Form-36 (SF-36). Tertiary endpoints included comparative safety at 1 year between the abatacept and infliximab groups.

### Clinical assessments

Disease activity was assessed using the validated DAS28 (ESR)*.*^8^ The EULAR criteria were used to assess good responses (endpoint DAS28 (ESR) of ⩽3.2 and an improvement from baseline of ⩾1.2).^6^ Signs and symptoms of RA were evaluated using ACR 20, 50 and 70 response rates based on the ACR criteria.^7^

Physical function was assessed using the HAQ-DI.^9^ Health-related quality of life (HRQoL) was assessed using the SF-36.^10^

### Safety

All patients who received at least one dose of study drug were evaluated for safety, including all reported AEs, serious AEs (SAEs), and clinically significant changes in vital signs, physical exams, and clinical laboratory test abnormalities.

#### Sample size calculation

The sample size and power were calculated to detect a treatment difference in the primary analysis of a mean change from baseline in DAS28 (ESR) for the abatacept vs placebo groups at day 197. Prospectively, this study was not powered with a superiority or non-inferiority design to compare the two active arms.

### Statistical analyses

All patients who received at least one dose of study medication were assessed for efficacy and safety (intent-to-treat population). At day 197, the abatacept or infliximab groups were compared with the placebo group by analyses of covariance (ANCOVA) for mean changes from baseline in DAS28 (ESR) and in the SF-36 (PCS and MCS). The model included the change as the dependent variable, with treatment group as a main effect and the baseline score as an additional covariate. The proportion of patients with ACR 20, 50 and 70 responses, LDAS, DAS28-defined remission, a good EULAR response and a clinical meaningful HAQ-DI response was calculated. The χ^2^ test was performed to evaluate the differences (and 95% CIs) between the abatacept or infliximab groups and placebo. At day 365, the reference group was changed to infliximab.

Patients who discontinued the study prematurely were considered as non-responders subsequent to the time of discontinuation for ACR 20, 50 and 70 responses, good EULAR responses and clinically meaningful HAQ-DI responses. For all continuous measurements (mean changes in DAS28, SF-36 and the HAQ-DI score), LDAS and DAS28-defined remission the last observations prior to the discontinuation were carried forward (LOCF). To access the effect of antirheumatic medications on the abatacept and infliximab treatment groups, a predefined sensitivity analysis was conducted on the data with the last DAS28 (ESR) score just prior to the initiation of the additional DMARD, or any increase in MTX or corticosteroid use during treatment days 198–365 carried forward.

Safety was assessed at each visit. Summary statistics were tabulated by treatment group at days 197 and 365.

## RESULTS

### Baseline demographics and clinical characteristics

**Figure 1:**
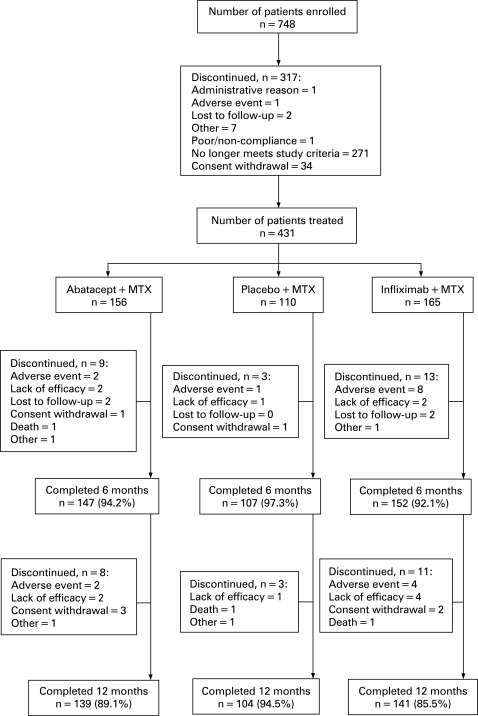
Patient disposition over 1 year. The ATTEST trial was a 12-month global trial conducted at 86 sites in the US (20 sites), Europe (18 sites (5 in Poland, 4 in Spain, 4 in Sweden, 2 in Russia, 2 in Denmark and 1 in Switzerland)), Canada (11 sites), Australia (6 sites), Mexico (10 sites), Argentina (5 sites), Brazil (8 sites), Peru (5 sites) and South Africa (3 sites). Patients were randomised in a 3:3:2 ratio to 6 months of abatacept (approximating 10 mg/kg), infliximab (3 mg/kg), or placebo treatment. During days 198–365, efficacy and safety data are not presented for the placebo group following reallocation to abatacept.

A total of 431 patients were randomised and treated with abatacept (n = 156), placebo (n = 110) or infliximab (n = 165) (fig 1). Baseline demographics and clinical characteristics were similar between groups (table 1). At randomisation, patients had active disease despite background MTX (mean DAS28 (ESR) of 6.8–6.9, tender joint counts 30.3–31.7, swollen joint counts 20.1–21.3 and mean HAQ-DI of 1.7–1.8). The mean dose of MTX was 16.3–16.6 mg and mean treatment duration was 18.3–23.7 months.

**Table 1:** Baseline demographics and clinical characteristics

Demographic/characteristic	Abatacept + MTX (n = 156)	Placebo + MTX (n = 110)	Infliximab + MTX (n = 165)
Age, years (SD)	49.0 (12.5)	49.4 (11.5)	49.1 (12.0)
Gender, % female	83.3	87.3	82.4
Race, % Caucasian	80.8	76.4	80.6
Geographic origin:			
North America, n (%)	16 (10.3)	10 (9.1)	15 (9.1)
South America, n (%)	93 (59.6)	66 (60.0)	96 (58.2)
Europe, n (%)	39 (25.0)	29 (26.4)	39 (23.6)
Rest of the world, n (%)	8 (5.1)	5 (4.5)	15 (9.1)
Disease duration, years (SD)	7.9 (8.5)	8.4 (8.6)	7.3 (6.2)
Tender joints, n (SD)	31.6 (13.9)	30.3 (11.7)	31.7 (14.5)
Swollen joints, n (SD)	21.3 (8.6)	20.1 (7.0)	20.3 (8.0)
Erythrocyte sedimentation rate, mm/h (SD)	49.4 (31.2)	47.0 (32.6)	47.8 (30.4)
C-reactive protein levels, mg/dl (SD)	3.1 (2.7)	2.7 (2.6)	3.3 (3.2)
DAS28 (ESR), n (SD)	6.9 (1.0)	6.8 (1.0)	6.8 (0.9)
HAQ-DI, 0–3 (SD)	1.8 (0.6)	1.8 (0.7)	1.7 (0.7)
Rheumatoid factor positive, n (%)	136 (87.2)	85 (77.3)	140 (84.8)
Concomitant medications			
Total patients on concomitant medications, n (%)	156 (100)	110 (100)	165 (100)
MTX, n (%)	156 (100)	110 (100)	164 (99.4)
Dose, mg/week (SD)	16.5 (3.7)	16.6 (3.7)	16.3 (3.6)
Duration, months (SD)	18.3 (20.0)	23.7 (25.6)	23.6 (26.8)
Corticosteroids, n (%)	118 (75.6)	77 (70.0)	118 (71.5)
NSAIDs, n (%)	133 (85.3)	93 (84.5)	142 (86.1)

MTX, methotrexate; DAS28 (ESR), Disease Activity Score 28 (based on erythrocyte sedimentation rate levels); HAQ-DI, Health Assessment Questionnaire Disability Index; NSAID, non-steroidal anti-inflammatory drug.

### Use of additional medications

Concomitant medications and NSAIDs were used by a similar proportion of patients across treatment groups at randomisation (table 1). Between days 198–365, when the protocol permitted adjustments to background medications, 12.8% and 17.6% of abatacept and infliximab-treated patients, respectively, added a DMARD, or increased their dose of MTX/corticosteroids from baseline.

### Discontinuations

During the first 6 months, discontinuations occurred in 5.8, 2.7 and 7.9% of the abatacept, placebo and infliximab groups, respectively. Between days 198–365, 5.1 and 6.7%, of the abatacept and infliximab groups, respectively, discontinued. Discontinuation due to AEs and SAEs were highest in the infliximab group in both periods (fig 1). Similar numbers of patients from the abatacept and infliximab groups completed 1 year of treatment (fig 1).

### Clinical efficacy

#### DAS28

**Figure 2:**
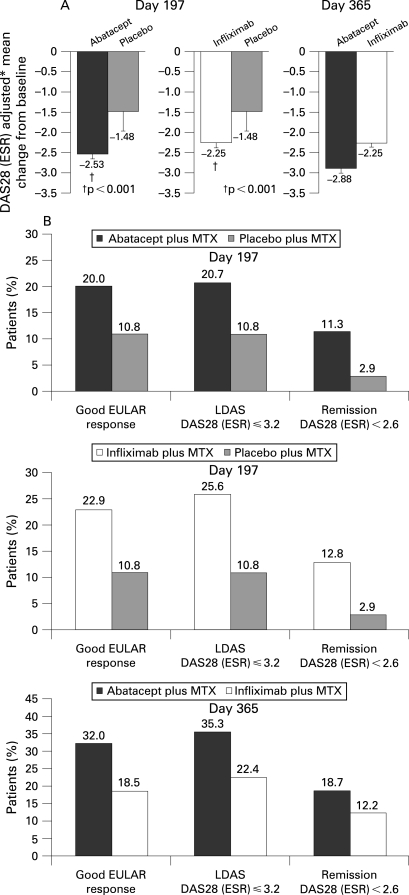
Disease Activity Score 28 (DAS28) based on erythrocyte sedimentation rates (ESR). A. DAS28 (ESR) mean changes from baseline at days 197 and 365. Error bars represent standard error of the mean. B. European League Against Rheumatism (EULAR) good responses, low DAS (LDAS; DAS28 ⩽3.2) and DAS28 (ESR)-defined remission at day 197 and at day 365. Data are presented for the intent-to-treat population with a last-observation carried forward analysis for mean changes in DAS28 (ESR), LDAS and DAS28 (ESR)-defined remission. Good EULAR responses were presented for the intent-to-treat population with patients who discontinued the study prematurely considered as non-responders subsequent to the time of discontinuation. Error bars show standard error of the mean. *Adjustment based on covariance with treatment as factor and baseline as covariant.

At day 197, the reduction in DAS28 (ESR), was significantly greater with abatacept vs placebo (–2.53 vs –1.48, p<0.001; fig 2A). A greater proportion of patients also experienced a good EULAR response (20.0 vs 10.8%), LDAS (20.7 vs 10.8%) and were in DAS28 (ESR)-defined remission (11.3 vs 2.9%), for abatacept vs placebo, respectively (fig 2B). Reductions in DAS28 (ESR) were also significantly greater in the infliximab vs placebo groups at day 197 (–2.25 vs –1.48, p<0.001; fig 2A), with a higher proportion of patients experiencing a good EULAR response (22.9 vs 10.8%), LDAS (25.6 vs 10.8%) and were in DAS28 (ESR)-defined remission (12.8 vs 2.9%), respectively (fig 2B). At day 197, the relative efficacy of abatacept and infliximab as assessed by the DAS28 (ESR) was similar.

At day 365, a greater reduction in DAS28 (ESR) was observed with abatacept than with infliximab (fig 2A; –2.88 vs –2.25; estimate of difference (95% CI) = –0.62 (–0.96, –0.29)). Also, the proportion of patients achieving a good EULAR response (32.0 vs 18.5%, estimate of difference (95% CI) = 13.5% (3.6, 23.3)), LDAS (35.3 vs 22.4%, estimate of difference (95% CI) = 12.9 (2.1, 23.7)), and DAS28 (ESR)-defined remission (18.7 vs 12.2%, estimate of difference (95% CI) = 18.7 (–2.2, 15.2)) were higher with abatacept compared with infliximab (fig 2B).

When disease activity was assessed using a sensitivity analysis (LOCF prior to increase in antirheumatic medications was performed), consistent improvements were demonstrated with abatacept: DAS28 (ESR) mean changes were significantly greater with abatacept vs infliximab at day 365 (–2.8 vs –2.2, respectively (difference from infliximab of –0.7, 95% CI of –1.0, –0.3)).

#### American College of Rheumatology response rates

**Figure 3:**
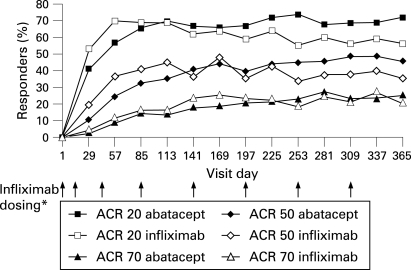
American College of Rheumatology (ACR) responses over 1 year. Proportion of patients with ACR 20, 50 and 70 responses was assessed on each visit day. Data are presented for the intent-to-treat population with a last-observation carried forward analysis. *Infliximab was administered on days 1, 15, 43, 85 and then every 56 days thereafter. Abatacept dosing occured at each visit day presentation following the assesment of efficacy.

At day 197, ACR 20, 50 and 70 responses were significantly greater with abatacept vs placebo (ACR 20: 66.7 vs 41.8%, p<0.001; ACR 50: 40.4 vs 20.0%, p<0.001; and ACR 70: 20.5 vs 9.1%, p = 0.019). ACR 20, 50 and 70 responses were also significantly higher in the infliximab group vs placebo (ACR 20: 59.4 vs 41.8%, p = 0.006; ACR 50: 37.0 vs 20.0%, p = 0.004; and ACR 70: 24.2 vs 9.1%, p = 0.002). The onset of response, as assessed by ACR 20 response rates, was generally more rapid for infliximab compared with abatacept (fig 3), however, by day 85, responses were similar (fig 3). Abatacept and infliximab demonstrated similar responses at day 197. During the second 6 months of the trial, the responses associated with abatacept were maintained, while those observed with infliximab were not. At day 365, ACR 20 responses were higher with abatacept than with infliximab (ACR 20: 72.4 vs 55.8%, difference of 16.7, 95% CI = 5.5, 27.8). In addition, the percentages of ACR 50 and 70 responders were numerically higher with abatacept vs infliximab treatment (with overlapping 95% CIs for the estimate of difference for ACR 50: 45.5 vs 36.4%, estimate of difference (95% CI) = 9.1 (–2.2, 20.5); ACR 70: 26.3 vs 20.6%, estimate of difference (95% CI) = 5.7 (–4.2, 15.6), respectively).

#### Physical function

At 6 months, significantly more patients in the abatacept group than in the placebo group demonstrated a clinically meaningful improvement in physical function (HAQ-DI responses: 61.5 vs 40.9%, respectively, p = 0.001). Similarly, significantly more patients in the infliximab group vs the placebo group achieved a HAQ-DI response (58.8 vs 40.9%, p = 0.005). At day 365, HAQ-DI responses were maintained in the abatacept and infliximab groups (57.7 and 52.7%, respectively, estimate of difference (95% CI) = 5.0 (–6.5, 16.5)).

#### Health-related quality of life

**Figure 4:**
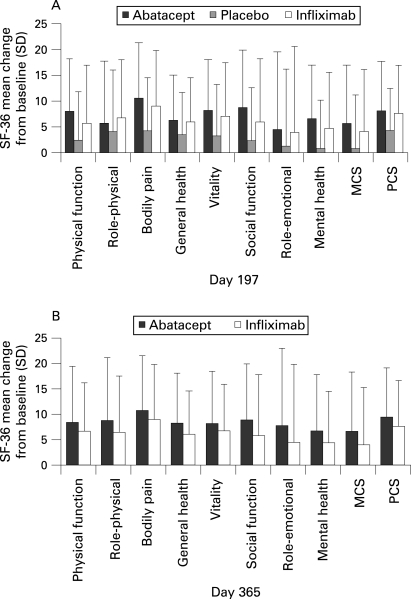
Health-related quality of life. A. Mean change in physical and mental component summary (PCS and MCS, respectively) scores and the individual subscales of the Short Form-36 (SF-36) from day 1 to day 197. B. Mean change in PCS and MCS scores and the individual subscales of the SF-36 from day 1 to day 365; Data are presented for the intent-to-to treat population with a last observation carried forward analysis.

Patients in the abatacept group experienced statistically significantly greater improvements from baseline in the PCS (p<0.001) and MCS (p = 0.004) of the SF-36, and in each of the eight individual subscales compared with placebo, following 6 months of treatment (fig 4A). Patients in the infliximab group also experienced significantly greater improvements from baseline in the PCS (p = 0.002) and MCS (p = 0.027), and all eight subscales of the SF-36 at day 197 compared with patients in the placebo group. At day 365, greater improvements from baseline in the PCS were observed with abatacept vs infliximab (difference of 1.93, 95% CI = 0.02, 3.84). Improvements in the MCS (difference of 1.92, 95% CI = –0.30, 4.15) and in all eight subscales were also numerically higher with abatacept vs infliximab (fig 4B).

### Safety

A summary of safety for all patients who received at least one dose of study medication is presented in table 2 (excluding the original placebo group between days 197–365).

**Table 2:** Summary of safety to day 197 and day 365

	Days 1–197			Days 1–365	
	Abatacept + MTX (n = 156)	Placebo + MTX (n = 110)	Infliximab + MTX (n = 165)	Abatacept + MTX (n = 156)	Infliximab + MTX (n = 165)
	n (%)*	n (%)*	n (%)*	n (%)*	n (%)*
Deaths	1 (0.6)	0	1 (0.6)	1 (0.6)	2 (1.2)
SAEs	8 (5.1)	13 (11.8)	19 (11.5)	15 (9.6)	30 (18.2)
Related SAEs	3 (1.9)	3 (2.7)	8 (4.8)	5 (3.2)	14 (8.5)
Discontinuations due to SAEs	2 (1.3)	0	4 (2.4)	4 (2.6)	6 (3.6)
AEs	129 (82.7)	92 (83.6)	140 (84.8)	139 (89.1)	154 (93.3)
Related AEs	64 (41.0)	46 (41.8)	74 (44.8)	72 (46.2)	96 (58.2)
Discontinuations due to AEs	3 (1.9)	1 (0.9)	8 (4.8)	5 (3.2)	12 (7.3)
Serious infections	2 (1.3)	3 (2.7)	7 (4.2)	3 (1.9)	14 (8.5)
Autoimmune symptoms and disorders	1 (0.6)	1 (0.9)	1 (0.6)	2 (1.3)	1 (0.6)
Malignant neoplasms	1 (0.6)	1 (0.9)	2 (1.2)	1 (0.6)	2 (1.2)

*More than one AE or SAE could be reported in each patient; percentages represent the proportion of patients who reported ⩾1 event.

AE, adverse event; MTX, methotrexate; SAE, serious adverse event.

#### Summary of safety for days 1–197

During days 1–197, AEs were reported by a similar proportion of patients in the abatacept (82.7%), placebo (83.6%) and infliximab (84.8%) groups. Two deaths were reported: one patient in the abatacept group due to a cerebrovascular accident, and one patient in the infliximab group due to fibrosarcoma. Overall, the frequency of AEs, related AEs and discontinuations due to AEs or SAEs was similar for the abatacept and placebo groups. Between days 1–197, SAEs were lower with abatacept vs placebo (5.1 vs 11.8%), the difference was largely attributed to a higher frequency of gastrointestinal disorders, bacterial infections and musculoskeletal disorders in the placebo group. For the infliximab vs placebo groups during the same period, the frequency of AEs (84.8 vs 83.6%), related AEs (44.8 vs 41.8%) and SAEs (11.5 vs 11.8%) were similar; however, a higher proportion of patients in the infliximab group compared with the placebo group reported related SAEs (4.8 vs 2.7%), discontinued due to AEs (4.8 vs 0.9%), and discontinued due to SAEs (2.4 vs 0%). The higher frequency of SAEs in the infliximab vs placebo groups was largely due to an increase in serious infections (4.2 vs 2.7%, respectively).

The most frequently reported AEs were primarily infections and infestations, and most were of mild to moderate intensity. Acute infusional AEs (within 3 h of the start of dosing) were reported in 5.1, 10.0 and 18.2% of the abatacept, placebo and infliximab groups, respectively (table 3). Infections and infestations were reported in 48.1, 51.8 and 52.1% of the abatacept, placebo and infliximab groups, respectively.

**Table 3:** Frequently occurring acute infusional events (⩾2.0% of patients in any group) to day 197 and day 365

Acute infusional events	Days 1–197			Days 1–365	
	Abatacept + MTX (n = 156)	Placebo + MTX (n = 110)	Infliximab + MTX (n = 165)	Abatacept + MTX (n = 156)	Infliximab + MTX (n = 165)
	n (%)	n (%)	n (%)	n (%)	n (%)
Total patients with AEs	8 (5.1)	11 (10.0)	30 (18.2)	11 (7.1)	41 (24.8)
Hypotension	0	0	7 (4.2)	0	8 (4.8)
Headache	2 (1.3)	2 (1.8)	7 (4.2)	2 (1.3)	7 (4.2)
Nausea	2 (1.3)	1 (0.9)	6 (3.6)	3 (1.9)	7 (4.2)
Flushing	1 (0.6)	0	4 (2.4)	1 (0.6)	5 (3.0)
Dyspnea	0	0	4 (2.4)	0	5 (3.0)
Urticaria	0	1 (0.9)	4 (2.4)	0	8 (4.8)
Pruritus	0	0	2 (1.2)	0	5 (3.0)
Dizziness	0	0	2 (1.2)	1 (0.6)	4 (2.4)

AE, adverse event; MTX, methotrexate.

Serious infections were lower in the abatacept group (1.3%) than in the placebo (2.7%) and infliximab (4.2%) groups at day 197 (table 4), with patients originating from Latin America (abatacept, 8.3%; placebo, 16.7%; infliximab, 25.0%) and Europe (abatacept, 8.3%; placebo, 8.3%; infliximab, 33.3%). Between days 1–197, two opportunistic infections occurred (a pseudomonal lung infection and a *Pneumocysitis jiroveci* pneumonia) in the infliximab group.

**Table 4:** Serious infectious events to day 197 and day 365

	Days 1–197			Days 1–365	
	Abatacept + MTX (n = 156)	Placebo + MTX (n = 110)	Inflixima + MTX (n = 165)	Abatacept + MTX (n = 156)	Infliximab + MTX (n = 165)
	n (%)	n (%)	n (%)	n (%)	n (%)
Total patients with SAEs	8 (5.1)	13 (11.8)	19 (11.5)	15 (9.6)	30 (18.2)
Infections and infestations	2 (1.3)	3 (2.7)	7 (4.2)	3 (1.9)	14 (8.5)
Pneumonia	2 (1.3)	0	2 (1.2)	2 (1.3)	3 (1.8)
Sinusitis	1 (0.6)	0	0	1 (0.6)	0
Postoperative wound infection	0	1 (0.9)	0	0	1 (0.6)
Soft tissue abscess	0	1 (0.9)	0	0	0
Infective bursitis	0	1 (0.9)	0	0	0
Bronchitis	0	0	1 (0.6)	0	1 (0.6)
Cellulitis	0	0	1 (0.6)	0	1 (0.6)
Gastroenteritis	0	0	1 (0.6)	0	1 (0.6)
Herpes zoster	0	0	1 (0.6)	0	1 (0.6)
Lung infection pseudomonal	0	0	1 (0.6)	0	1 (0.6)
*Pneumocystis jiroveci* pneumonia	0	0	1 (0.6)	0	1 (0.6)
Infection skin ulcer	0	0	0	1 (0.6)	0
Encephalitis herpetic	0	0	0	0	1 (0.6)
Erysipelas	0	0	0	0	1 (0.6)
Lobar pneumonia	0	0	0	0	1 (0.6)
Peritoneal tuberculosis	0	0	0	0	1 (0.6)
Pulmonary tuberculosis	0	0	0	0	1 (0.6)
Septic shock	0	0	0	0	1 (0.6)

MTX, methotrexate; SAE, serious adverse event.

Autoimmune symptoms or disorders were uncommon (<1%), occurring at a similar frequency across groups (table 2). Malignancies were reported for four patients, including one abatacept-treated patient (bladder cancer), one placebo-treated patient (non-melanomatous skin cancer) and two infliximab-treated patients (malignant anorectal neoplasm and fibrosarcoma in one patient for each).

#### Summary of safety for days 1–365 for abatacept and infliximab groups

During the entire 12-month, double-blind period (days 1–365), SAEs, related SAEs, and discontinuations due to SAEs were lower with abatacept than infliximab (SAEs: 9.6 vs 18.2%; related SAEs: 3.2 vs 8.5%; and discontinuations due to SAEs: 2.6 vs 3.6%, respectively (table 2)). Overall, AEs, related AEs and discontinuations due to AEs were also lower with abatacept than with infliximab (AEs: 89.1 vs 93.3%; related AEs: 46.2 vs 58.2%: and discontinuations due to AEs: 3.2 vs 7.3%, respectively). An additional infliximab-treated patient with peritoneal TB died during the second 6 months of the trial due to septic shock following surgery. One patient who was randomised to the placebo group died while receiving abatacept between days 198–365 from pneumonia and sepsis. The investigator assessment deemed the death possibly related to study treatment.

Acute infusional events (7.1 vs 24.8%; table 3) were lower with abatacept vs infliximab, respectively.

Up to day 365, infections and infestations were reported in 59.6 and 68.5% of the abatacept and infliximab groups, respectively. Serious infections were reported in 1.9% of abatacept-treated patients and 8.5% of infliximab-treated patients (table 4). A total of five serious opportunistic infections were reported, all occurring in the infliximab group in one patient for each (encephalitis herpetic, lung infection pseudomonal, peritoneal TB, *P jiroveci* pneumonia and pulmonary TB). Both cases of TB were in patients who were PPD test negative and chest *x* ray negative at study entry. Between days 1–365, autoimmune symptoms or disorders were uncommon (<1%) and occurred at a similar frequency in the abatacept and infliximab groups (table 2). No additional malignant neoplasms were reported in either group between days 198–365.

## DISCUSSION

The multi-centre, double-blind, placebo-controlled ATTEST trial, examining the relative efficacy and safety of abatacept or infliximab vs placebo, confirms that both biologics are effective for the treatment of RA. Over the 12-month study, a relative difference in safety was observed, with fewer SAEs, serious infections, acute infusional events and discontinuations due to AEs in the abatacept group than the infliximab group.

Following 6 months of treatment, abatacept and infliximab on a background of MTX significantly reduced the signs and symptoms of disease (DAS28 (ESR); ACR 20, 50 and 70), and improved physical function (HAQ-DI) and quality of life (SF-36) compared with placebo, in patients with an inadequate response to MTX. The relative efficacy of abatacept and infliximab at day 197 was similar, as the 95% CIs for the treatment difference for DAS28 (ESR) scores, ACR response rates, HAQ-DI responses and HRQoL improvements overlapped. However, by day 365, the 95% CIs for the treatment difference between abatacept and infliximab for the reduction in DAS28 (ESR), good EULAR responses, LDAS, the ACR 20 response rate and the HRQoL physical summary measure did not include zero, suggesting a difference favouring abatacept for these specific endpoints.

While the onset of response (as assessed by ACR 20 responses), initially appeared more rapid with infliximab, similar response rates were noted with abatacept and infliximab by day 85. Between 6 months and 1 year, abatacept responses were maintained, while those with infliximab were not. The sustained efficacy observed at 1 year compared with 6 months in abatacept-treated patients is consistent with results from other abatacept trials.^2^ ^3^ Similarly, the finding that patients treated with infliximab did not sustain response rates over 1 year is also consistent with previous trials.^11^ When the impact of adding additional therapies was assessed using a sensitivity analysis according to the last score prior to addition, reductions in disease activity tended to be consistently higher with abatacept than with infliximab.

Overall, abatacept had a relatively more acceptable safety and tolerability profile than infliximab. Over 1 year, fewer SAEs, AEs, infections, and discontinuations due to AEs or SAEs were observed with abatacept relative to infliximab. Generally, opportunistic infections appeared to be relatively uncommon in this study and were mainly observed in the infliximab group, including two events of TB, an event of *P jiroveci* pneumonia, an event of pseudomonal lung infection and an event of herpes encephalitis. No opportunistic infections were reported in the abatacept group. Autoimmune symptoms or disorders were uncommon (<1%) with abatacept and infliximab. All of the malignancies were reported during the first 6 months, including one in the abatacept group, one in the placebo group and two in the infliximab group.

The data presented here should be interpreted within the context of several limitations. Although collectively the data support a relatively more acceptable risk-to-benefit profile of abatacept compared with infliximab, the design of this study utilised the infliximab dose of 3 mg/kg, without dose adjustment. At the time of the study, the recommended dose of infliximab (approved labelled starting dose for RA) was 3 mg/kg; however, today regulatory agencies recognise the use of higher doses of infliximab, and physicians use them in a proportion of patients to maintain a durable response. In fact, the dose may be increased in up to 30% of patients.^12^ Although higher doses have been associated with an increased risk of AEs in clinical trials.^5^ ^13^ Finally, this study was not designed to evaluate the effect of abatacept or infliximab vs placebo on radiographic progression.

In conclusion, these two biologic therapies with two distinct mechanisms of action have different safety and efficacy profiles; however, abatacept and infliximab both offer clinical improvements to patients with an inadequate response to MTX. Over 1 year, abatacept exhibited a durable response, and had a relatively more acceptable safety and tolerability profile than infliximab, as evidenced by fewer SAEs, serious infections, acute infusional events and discontinuations due to AEs.

## References

[1] Yamada A Salama AD Sayegh MH The role of novel T cell costimulatory pathways in autoimmunity and transplantation. J Am Soc Nephrol 2002;13:559–75 11805188 10.1681/ASN.V132559

[2] Kremer JM Genant HK Moreland LW Russell AS Emery P Abud-Mendoza C Effects of abatacept in patients with methotrexate-resistant active rheumatoid arthritis: a randomized trial. Ann Intern Med 2006;144:865–76 16785475 10.7326/0003-4819-144-12-200606200-00003

[3] Genovese M Becker J-C Schiff M Luggen M Sherrer Y Kremer J Abatacept for rheumatoid arthritis refractory to tumor necrosis factor alpha inhibition. N Engl J Med 2005;353:1114–23 16162882 10.1056/NEJMoa050524

[4] Arnett FC Edworthy SM Bloch DA McShane DJ Fries JF Cooper NS The American Rheumatism Association 1987 revised criteria for the classification of rheumatoid arthritis. Arthritis Rheum 1988;31:315–24 3358796 10.1002/art.1780310302

[5] Maini RN Breedveld FC Kalden JR Smolen JS Davis D Macfarlane JD Therapeutic efficacy of multiple intravenous infusions of anti-tumor necrosis factor alpha monoclonal antibody combined with low-dose weekly methotrexate in rheumatoid arthritis. Arthritis Rheum 1998;41:1552–63 9751087 10.1002/1529-0131(199809)41:9<1552::AID-ART5>3.0.CO;2-W

[6] Fransen J van Riel PL The Disease Activity Score and the EULAR response criteria. Clin Exp Rheumatol 2005;23 5 Suppl 39:S93–9 16273792

[7] Felson DT Anderson JJ Boers M Bombardier C Furst D Goldsmith C American College of Rheumatology. Preliminary definition of improvement in rheumatoid arthritis. Arthritis Rheum 1995;38:727–35 7779114 10.1002/art.1780380602

[8] Prevoo ML van’t Hof MA Kuper HH van Leeuwen MA van de Putte LB van Riel PL Modified disease activity scores that include twenty-eight-joint counts. Development and validation in a prospective longitudinal study of patients with rheumatoid arthritis. Arthritis Rheum 1995;38:44–8 7818570 10.1002/art.1780380107

[9] Wells GA Tugwell P Kraag GR Baker PR Groh J Redelmeier DA Minimum important difference between patients with rheumatoid arthritis: the patient’s perspective. J Rheumatol 1993;20:557–60 8478873

[10] Samsa G Edelman D Rothman ML Williams GR Lipscomb J Matchar D Determining clinically important differences in health status measures: a general approach with illustration to the Health Utilities Index Mark II. Pharmacoeconomics 1999;15:141–55 10351188 10.2165/00019053-199915020-00003

[11] Lipsky PE van der Heijde DM St Clair EW Furst DE Breedveld FC Kalden JR Infliximab and methotrexate in the treatment of rheumatoid arthritis. Anti-Tumor Necrosis Factor Trial in Rheumatoid Arthritis with Concomitant Therapy Study Group. N Engl J Med 2000;343:1594–602 11096166 10.1056/NEJM200011303432202

[12] Rahman MU Strusberg I Geusens P Berman A Yocum D Baker D Double-blinded infliximab dose escalation in patients with rheumatoid arthritis. Ann Rheum Dis 2007;66:1233–8 17392352 10.1136/ard.2006.065995 PMC2652128

[13] Westhovens R Yocum D Han J Berman A Strusberg I Geusens P The safety of infliximab, combined with background treatments, among patients with rheumatoid arthritis and various comorbidities: a large, randomized, placebo-controlled trial. Arthritis Rheum 2006;54:1075–86 16572442 10.1002/art.21734

